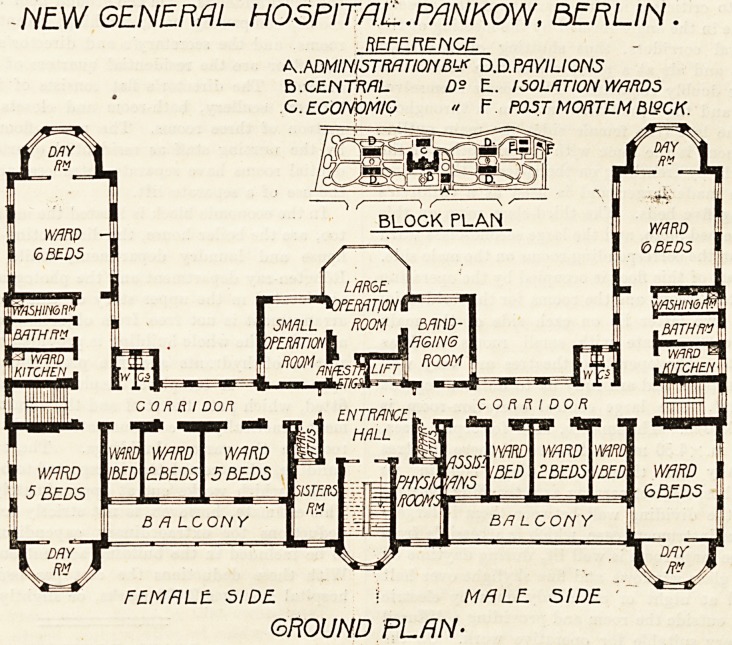# Some Modern Continental Hospitals

**Published:** 1909-01-30

**Authors:** 


### HOSPITAL ADMINISTRATION.

### CONSTRUCTION AND ECONOMICS.

### SOME MODERN CONTINENTAL HOSPITALS.

THE NEW GENERAL HOSPITAL AT PANKOW, BERLIN.

This institution, which adequately serves the needs of a rising residential suburb of Berlin, may be looked upon as one of the best and most up-to-date of the smaller general hospitals in Germany. The plans, drawn up by Herr Wilhelm Johow, provide for efficient accommodation for 200 patients, and though the hospital has not yet been entirely completed, the main part of it is already occupied, and from its inspection a good idea of the whole institution can be gained.

As will be seen from the block plan annexed the institution is built on the modified pavilion system, the main feature being the large central block, which is a corridor building. On each side of this block lie two pavilions; behind is the economic block, and to the left, anterior to the main building, is the administrative block. On the extreme left are two isolation barracks and the pathological building. The buildings are situate in a well laid-out park, which affords convalescents ample opportunity for enjoying open air.

The main block is designed on the corridor system, the corridors being so planned as to give the wards triple-sided lighting and ventilation openings. It lies with its long axis so directed that the wards face north and south. The wards themselves are small, and designed to accommodate from three to six patients each. On the ground-floor are the entrance-hall and vestibule, the main nurses' hall, a room for apparatus and special instruments, a dispensary, and the residential quarters of the assistant physicians. Behind these is a well-equipped hydro-therapeutic department with the necessary addenda in the shape of waiting, dressing, massage, and recuperating rooms. In the side wings on the ground floor are the separate wards. The arrangement of these wards is better seen from the first-floor plan, which we append, the arrangement being identical with that which obtains on the ground floor. On the left side lies the department for male, on the right that for female patients, and the two divisions exactly correspond in details of construction, arrangement, and equipment. Thus, the main corridor, which stretches in the form of two rectangles placed at right angles to one another, from the one extremity of the wing to the middle of the centre block, is wide and roomy, adequately lighted and ventilated, the windows being large, wide-paned, and reaching well down to the floor. From this corridor one enters four wards, lying parallel to each other and at right angles to the main part of the corridor. Each ward has a fine balcony communicating with the one alongside it, so that there is really one large balcony available for the use of patients. The first ward is designed for what are called first-class patients—that is to say, for patients who pay a relatively higher daily sum for attendance and treatment than the other, or second-class patients. This room, which contains but one bed, is simply furnished, as in most private clinics, and is remarkable only for its excellent ventilation and the perfect lighting which has been provided for. The three remaining wards, for second-class patients, are of different dimensions. The middle and smallest one contains only one bed, and is very similar to the first-class private room; that on the left provides accommodation for two and the other for four patients. In all these wards the ventilation and lighting are admirable. The floors are of cement overlaid with thick linoleum. Artificial light is provided for by numerous electric lamps, while the whole of the central block is heated by means of a specially-designed system of hot-water pipes. During the summer months the windows, which are double, serve adequately for ventilation purposes. Proceeding up the corridor the visitor enters the wing which contains three small rooms—one used as the bath-room, the other as ward kitchen, and the third as utensil-room. Finally comes the end ward, for third-class patients, five of whom can be accommodated, and the large day-room, built in the form of a half circle with large alcove windows. This ward is one of the finest hospital wards we have seen, extravagantly capacious and admirably fitted. The bath-room is lined throughout with tiles and contains warm and cold baths and douches. The one feature of the arrangements of this part of the building which is open to criticism is the position of the closets. These are situate in the angle formed by the meeting of the wing and central corridors, thus shutting off a certain amount of light and air at a point where free ventilation and lighting are doubly requisite. The closets themselves are very good, and the ventilation in them is throughout efficient. On the left (the female side) the main outline of the arrangement is the same with the exception of the first ward, which, by trenching on the space of the central block, has been made larger and is used as a children's ward, containing five beds. The third-class room on this side contains one bed more, and the large second-class ward one bed less, than the corresponding rooms on the male side.

The central part of this floor is occupied by the operating theatres, the sisters' room, and the rooms for the residential assistant. The two latter lie on each side of the main stairway, and communicate with small rooms used as apparatus closets. The operating theatres are very well suited for the purpose, and are fully up to date as regards their arrangement. The large central operation-room is 5 m.×7.50 m.×4.30 m. The smaller, used for septic cases, is 4.60 m.×5.50 m.×4.30 m. A feature of these theatres (which are really not theatres as they contain no auditorium, the hospital not serving as a teaching theatre) is the fact that the dividing wall between them is largely composed of a fine instrument case, which is accessible from both rooms. The large room is well lit, during daytime by the large, open, glass windows and fine skylight over half of its roof; and at night or on cloudy days by electric arc lamps placed outside the room and providing a diffused light which is very suitable for operative work. On the right side of this large operating-room is a room which is used as an anæsthetising room. The operating-rooms are lined with porcelain tiles, the doors and all the iron and wood work being enamelled grey green. Hot water and sterile saline solution apparatus are provided, and the operating tables, though of exceedingly simple construction, are adequate and permit of all necessary movements required to alter the position of the patient.

On the second floor are two special rooms designed for the use of patients suffering from genito-urinary diseases or for those who cannot very well be kept in the general ward below. Here also, under the roof, are special rooms for light, air and sun baths. In conclusion, the building, which is built in a simple but dignified style, contains two automatically-working electric lifts, while drainage is adequately provided for, the drains opening into the main city canalisation system. In the basement are the laboratories and the closets for dirty linen. The other buildings comprise the administrative block, the economic block the pavilions and the isolation and pathological block. Owing to the fact that in a small general hospital the isolation department is usually not much required, while in times of local epidemic even a comparatively large isolation block is insufficient to deal adequately with all the cases, it has been decided to limit the isolation department to one simple pavilion which provides accommodation, in four isolated divisions, for three patients in each. These divisions are designed to serve for cases of scarlet fever, diphtheria, measles and typhus. The pavilion contains in addition a small operating-room, a large bath-room, and a ward kitchen and waiting-room. The four pavilions for ordinary patients, planned to the right and left of the main building, have not as yet been completed. They are designed to accommodate about twenty patients each, and will be provided with the usual day and work rooms. The administrative block comprises a basement, a ground, upper, and a first floor. On the basement floor are the cellarage, rooms for delirious and epileptic patients, and the usual addenda. On the ground floor are the receiving-rooms, comprising bath, clothing, reception and waiting rooms, and the secretary's and director's offices. On the first floor are the residential quarters of the director and matron. The director's flat consists of four rooms, with kitchen, scullery, bath-room and closets. That of the matron of three rooms. The upper floor rooms are used by the nursing staff as residential quarters. These residential rooms have separate entrances, and the staff have the use of a separate lift.

In the economic block is located the main kitchen. Here too, are the boiler house, the disinfecting-rooms, the wash-house and laundry department, while temporarily the Röngten-ray department and the photographic department are located in the upper story of this block. This latter arrangement is not free from objections and will soon be altered. The whole building is provided with an excellent system of hydrants and fire prevention and precaution apparatus. A complete telephone installation has been fitted, which puts the staff and the administrative department practically in communication with every ward and room in the various buildings. The total cost of the building, when completed, is expected to be about a million marks, which works out at approximately £230 per bed. This estimate, however, is not strictly correct, as various deductions for extraordinary expenditure not properly to be included in the building account ought to be made. With these deductions the cost per bed of this model hospital averages 2,000 marks, or slightly below £100.

## Figures and Tables

**Figure f1:**